# The Influence of the Terminal Phosphorothioate Diester Bond on the DNA Oxidation Process. An Experimental and Theoretical Approach

**DOI:** 10.3390/molecules200712400

**Published:** 2015-07-08

**Authors:** Boleslaw T. Karwowski

**Affiliations:** Food Science Department, Medical University of Lodz, ul. Muszynskiego 1, 90-151 Lodz, Poland; E-Mail: Bolek.Karwowski@wp.pl; Tel.: +48-42-677-9141

**Keywords:** phosphorothioate, DNA damage, UVA radiation, oxidation process, charge transfer

## Abstract

In this study, the influence of the terminal phosphorothioate (PT) internucleotide bond in *ds*-DNA on the oxidation process was taken into consideration. The interaction of UV with the targeted oligonucleotide leads to an electron ejection and radical cation “hole” migration through the *ds*-DNA until it is trapped irreversibly in a suitable place. Phosphorothiate internucleotide bonds were detected in the bacterial genome; however, their role is still unclear. In this study a PAGE analysis of irradiated *ds*-DNA showed that the degradation rea ction was slowed down by the presence PT next to the anthraquinone moiety. Further, theoretical study shows that [*R*_P_] AQ-PS-dG can adopt a slightly lower ionisation potential energy and triplet excited state with a subsequent slightly higher adiabatic electron affinity value in comparison with [*S*_P_] AQ-PS-dG and AQ-PO-dG. Moreover, the energy gap between HOMO and LUMO, indicated the radical stabilisation properties of [*R*_P_] AQ-PS-dG, which can hinder the charge transfer through *ds*-DNA.

## 1. Introduction

**Scheme 1:**
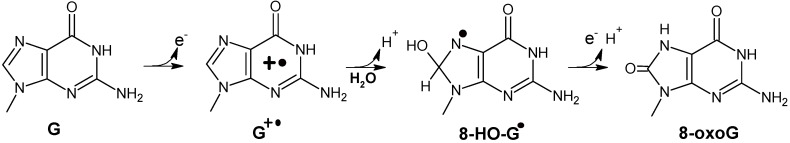
The formation of 8-oxoG in the one-electron oxidation process [6].

Genetic information is preserved in cellular DNA in every living organism [1]. This storage medium is continuously exposed to oxidative stress, which can lead to a variety of DNA lesions [2]. So far, more than 80 such DNA lesions have been detected [3,4]. One of the main causes of their ocurrence is the activity of reactive oxygen species (ROS), which may be generated by endocellular processes, such as the Haber-Weis type reaction [5]. Physical factors, such as ionisation radiation (gamma, X-rays, *etc.*) or solar light (95% UVA), can also induce the ROS formation process. Due to the nature of *ds*-DNA, in favourable conditions, once it has been oxidized, the formed radical cation “hole” can hop reversibly through the double-helix until it is trapped, usually by the reaction of the formed guanine radical cation (G^●+^) with H_2_O (Scheme 1) [6]. Depending on the nature of the G:::C base pair radical, two reaction paths are possible with or without the addition of a water molecule [6,7]. The first path leads to the 8-oxo-7,8-dihydroguanine (8-oxoG) formation.

**Figure 1:**
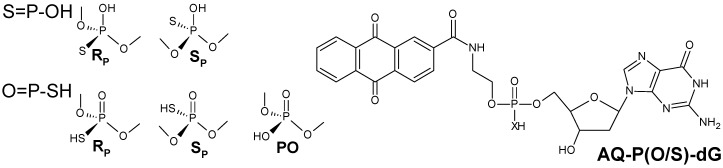
Graphical representation of AQ-PO-dG, AQ-PS-dG and phosphorothioate internucleotide bond stereochemistry and antraquinone (AQ) covalent linkage to 5′-end of the oligonucleotides.

On the other hand, in 2006 Schuster demonstrated that spermine disulphide can protect *ds*-DNA from oxidative damage [8]. One year later, in 2007, Wang *et al.* showed that phosphorothiate (PT) modification in an internucleotide bond can naturally appear in the bacterial genome [9]. This modification was not detected in other cells of living organisms. In phosphorothioate diester linkage, one of the non-bridging oxygen atoms is replaced by sulphur; this change leads to a new chiral centre appearing on the phosphorus atom (Figure 1). Due to the minimal distortion of the spatial geometry and π stacking interaction in *ds*-DNA, S-oligomers (S-DNA) have been widely studied as useful tools in antisense strategy [10,11]. In this work, the influence of the phosphorothioate internucelotide bond on the light-induced oxidation process was examined.

## 2. Results and Discussion

Among the variety of photo-oxidants, an anthraquinone (AQ) derivative covalently linked to the 5′-end was used to initiate the DNA strand cleavage [12,13]. To elucidate the influence of terminal PT on the DNA oxidation process two *ds*-DNA oligonucleotides (Table 1) were synthesized, purified and characterized according to the commonly followed procedure. Each of the duplexes contained an AQ moiety on the 5′-end of one strand and ^32^P on the 5′-end of the complementary one. In *oligo*-A the anthraqunione moiety was connected to a DNA strand by a phosphate diester bond, and in *oligo*-B by phosphorothioate one. Following the mechanisms proposed by Schuster, the oligonucleotides were irridiated in their double-stranded form by UVA (350 nm) and rapidly formed a triplet excited state of anthraquinone (AQ*^3^) with one-electron DNA oxidation [12,13]. Subsequently, the generated radical ion pair AQ^●─^B^●+^ could give rise toO_2_^●─^ formation by the reaction with oxygen. Therefore, it was possible for an individual nucleobase radical cation (B^●+^) to migrate through *ds*-DNA until it was annihilated [13].

**Table 1:** Structure/nucleobase sequence of *ds*-oligonucleotides with phosphorotioate linkage indication.

***oligo*-A**	**^32^P-5′**-^1^A^2^A^3^A^4^T^5^T^6^A^7^A^8^T^9^A^10^T^11^G^12^T^13^A^14^T^15^T^16^G^17^T^18^A^19^T^20^A^21^T^22^A^23^A^24^A^25^T^26^T^27^A^28^T^29^T-3′-**End**
	T T T A A T T A T A C A T A A C A T A T A T T T A A T A A-**AQ-5′**
***oligo*-B**	**^32^P-5′**-^1^A^2^A^3^A^4^T^5^T^6^A^7^A^8^T^9^A^10^T^11^G^12^T^13^A^14^T^15^T^16^G^17^T^18^A^19^T^20^A^21^T^22^A^23^A^24^A^25^T^26^T^27^A^28^T^29^T-3′-**End**
	T T T A A T T A T A C A T A A C A T A T A T T T A A T A A-_PS_-**AQ-5′**

Both double-stranded oligonucleotides, presented in Table 1, were irradiated at 350 nm for 0, 30, 60 and 120 min. The alkali-labile DNA sites/lesions were detected by an autoradiogram of PAGE analysis with the previous piperidine sample treatments. It is important to mention here that the profile of CD spectra as well as the Tm values assigned after 120 min of UVA irradiation showed the same profile and similar values as those denoted without radiation (data presented in supplementary materials). Moreover, as shown in the preparation of 5′-end-labeled oligonucleotides in the Supplementary Materials, no strand cleavages were observed before the piperidine treatment. These results indicated the absence of a hydroxyl radical (•OH). The activity of •OH can lead to an accidental-single strand break formation by abstraction of the hydrogen atom from the sugar moiety with subsequent 2-deoxyribose radical rearmament [5]. Additionally, the integrity of oligo-A and oligo-B after UVA irradiation was confirmed by a comparison of *ds*-DNA melting temperature values and CD spectra profiles with un-irradiated ones. These comparative analyses barely reveal any fluctuation for both discussed double-stranded oligonucleotides (see supplementary materials).

**Figure 2:**
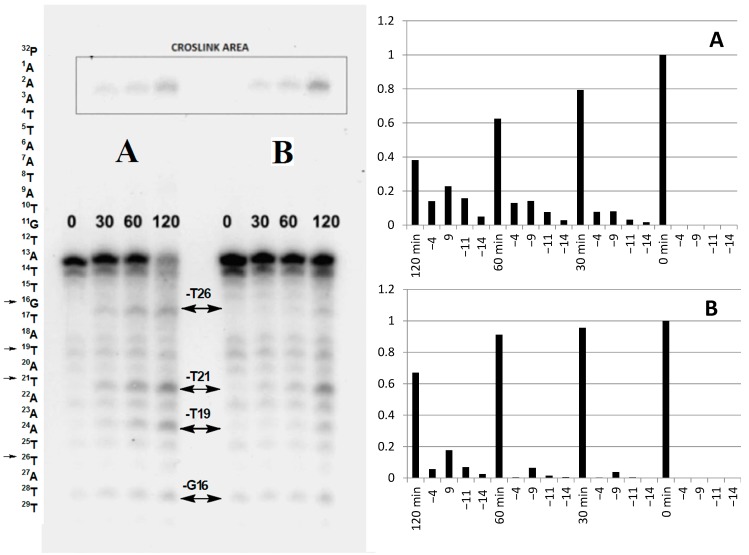
Autoradiogram of the results of irradiation of AQ-linked *oligo*-A (**A**) and *oligo*-B (**B**) and standard cleavage ratios of the investigated double-stranded oligonucleotides. Irradiation time 0, 30, 60, 120 min, -4 corresponding to T26, -9 to T21, -11 to T19, -14 to G19 in the sequence of ^32^P radiolabelled strand.

Based on the available literature data, the formation of DNA damage induced by a “hole” migration process was expected with its potential inhibition by the phosphorothioate moiety [8]. The distance between the AQ unit and the first guanine (G16) in this study was around 54 Å (*i.e.*, 14 bases); in Schuster’s previous work, this was extended up to 22 bases [14]. For this reason, a significant influence on 8-oxoG formation in the *oligo*-A was hypothetically expected, induced by a one-electron oxidising mechanism. Surprisingly, as depicted in Figure 2 for both *oligo*-A and *oligo*-B no strand cleavages were observed at the G16 position and hardly any at G11. Lesions were observed at the T19, T21, T26 locations. Additionally for both *ds*-oligos, intrastrand crosslink bands were denoted (Figure 2). The densitometry quantification of the ^32^P radio-labelled oligos PAGE analysis disclosed the influence of the PT internucleotide bond on *ds*-DNA’ stability. These unexpected results can be derived from the effect of the neighbouring base next to G. The effective energy of the positive charge localised in the middle of G in the trimer (5′-XGY-3′ sequence) was strongly dependent on enclosed nucleobases and adopts values in the range of 7.89 to 8.85 eV [15]. The highest values were assigned for 5′-TGC-3′ and 5′-TGT-3′. In the structure of both discussed oligonucleotides, two isolated Gs were flanked by thymidines. Due to that, the charge migration process, hypothetically, can be hindered. Additionally Giese noted, that the charge transfer from G to G, which are separated by a tetramer, occurs by a hopping mechanism, not tunnelling [16]. Based on above, the oxidation efficiency of G in the investigated *ds*-oligonucleotides, of G^11^ should be reduced.

However; on the other hand; in the case of PS-oligo; the difference in oligonucleotide resistance to UVA irradiation might be due to the dynamic fluctuation of the terminal 5′-end part of double-stranded DNA; which is slightly disturbed by the presence of PT. This can be supported by the fact that the PT linkage was positioned exactly on the 5′-end of the strand; where the stability of duplex is lower than in the central part of *ds*-oligo. Additionally; following the results of other authors; phosphorothioate can be recognized as an antioxidant [17]. This observation was indirectly supported when; during a negative electrospray ionization mass spectroscopy induced by a hydroxyl radical; a desulphurization process was noticed [18]. The “protective” role of the PT moiety against one-electron DNA oxidation was investigated for the first time by Sevilla *et al.* [19].

In this study, a comparison of the lesion patterns as a function of time revealed a difference between *oligo*-A and *oligo*-B (Figure 2). O*ligo-*B showed a higher UVA stability. In *ds*-DNA, *oligo*-B, the phosphorothioate diester bond shifted to the opposite strandin relation to oligo-A, and directly next to the AQ moiety. In this case, the cation radical migration through *ds*-DNA is potentially slowed down or inhibited. One hypothetical explanation of this phenomenon is that firstly the desulphurization process via •OH-induced S to O replacement of the phosphorothioate internucleotide bond, as described for ESI experiments [18], with a subsequent “hole” migration process, can take place. A second possible explanation of the discussed difference between A and B *ds*-DNA is the influence of PT, depending on phosphorus stereochemistry *R*_P_ or *S*_P_, on the spatial arrangement of AQ unit *vs.* duplex.

**Figure 3:**
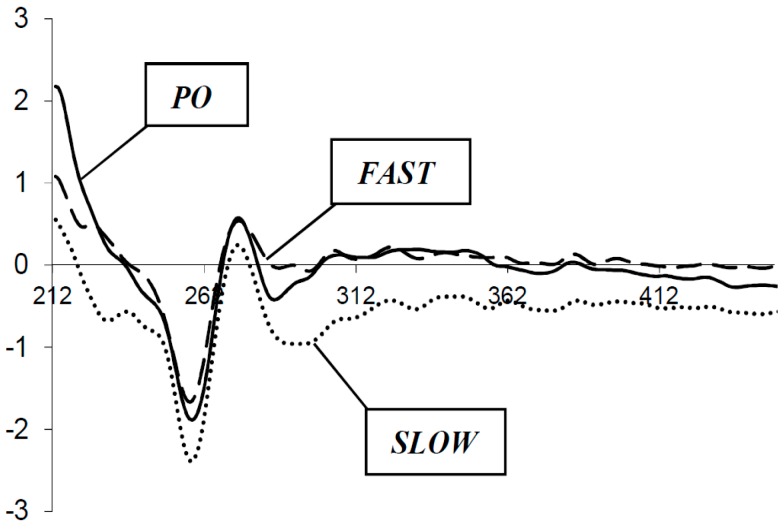
CD spectra of AQ-PO-dG, Fast and Slow forms of AQ-PS-dG molecules.

To elucidate the above postulate the AQ-PS-dG and AQ-PO-dG were synthesized (Scheme 1). The CD spectra of these model molecules, after their separation to pure diastereoisomeric forms *Fast* and *Slow*, showed differences in the regions of B DNA form (260 nm) and anthraquinone (300–400 nm). *Fast* is a form of the diastereomer with a shorter retention time under RP-HPLC analysis. Based on the previous results concerning the stereochemistry of phosphorothioates and Cahn-Ingold-Prelog roles, the *S*_P_ configuration of the phosphorus atom can be proposed for a “Fast” isomer and *R*_P_ for “Slow”. The CD spectra (Figure 3) of the *Fast* profile are much more similar to those of AQ-PO-dG than *Slow*. This observation suggests that the PT diastereomer corresponding to the *Fast* form is spatially similar to the construct with a natural phosphate bond. This can be supported by theoretically assigned—DM dipole moment values (Table 2). The [*S*_P_] AQ-PS-dG shows a higher DM among all the molecules in O=P-SH form, *i.e.*, 15.05[D]. This value is higher than for AQ-PO-dG by 2.42[D] and by 1.69[D] for [*R*_P_] AQ-PS-dG. For the S=P-OH variant the situation is opposite to the *R*_P_ diastereomer of AQ-PS-dG, which shows a higher DM value, *i.e.*, 16.89[D]. Surprisingly [*S*_P_] AQ-PS-dG and AQ-PO-dG adopted almost the same value *i.e.*, 12.90[D] and 12.63[D], respectively.

**Figure 4:**
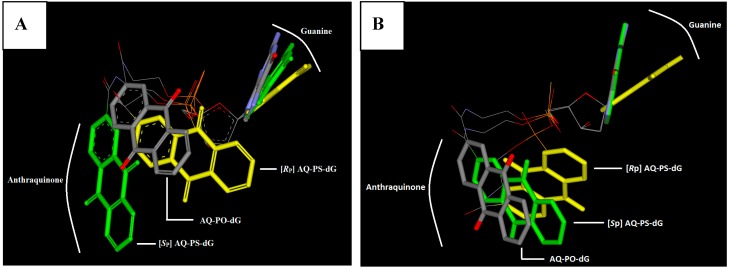
A spatial geometry comparison between AQ-PO-dG colored by elements, [*R*_P_] AQ-PS-dG—yellow and [*S*_P_] AQ-PS-dG—green after pentose ring overlap. The structures have been optimized at the M06-2X/6-31+G(d,p) level of theory in the aqueous phase in different protonated forms: (**A**) O=P-SH and (**B**) S=P-OH.

The DM differences that were found can force differences in the profile of CD spectra due to the fact that the dipole moment is involved in the Cotton effect [20]. Moreover, a comparison of the 3-D structures of the discussed molecules reveals a different spatial arrangement between the AQ moiety and guanine, as shown in Figure 4.

Therefore, further study of the absolute configuration on the phosphorus atom (crystallography or NMR) in the above mentioned molecules is highly recommended. Also, it can be postulated that the spatial position of the sulphur atom in the phosphodiester bond is crucial for the initiation of the hole hopping process in the case of *oligo*-B.

**Table 2:** The electronic properties of [*R*_P_] AQ-PS-dG, [*S*_P_] AQ-PS-dG, AQ-PO-dG obtained by calculation at M06-2X/6-31+G(d,p) and TD M06-2X/6-31++G(d,p) levels of theory in the aqueous phase, see Figure S13 (S—Supplementary Materials). Energy values given in eV and dipole moment (DM) in Debyes.

Electronic Properties	M06-2X/6-31+G(d,p)				
		AQ-PS-dG			
	AQ-PO-dG	[*R*_P_] Slow		[*S*_P_] Fast	
		O=P-SH	S=P-OH	O=P-SH	S=P-OH
**AIP**	5.96	5.81	5.84	6.03	5.99
**AIP^ZPVE^**	5.72	5.58	5.58	5.84	5.78
**VIP**	9.43	9.38	6.33	9.55	6.39
**VEAC**	3.90	3.88	5.67	3.81	5.66
**AEA**	3.68	3.79	3.84	3.69	3.68
**AEA^ZVPE^**	3.39	3.50	3.58	3.39	3.39
**VEA**	0.02	0.083	3.43	0.068	3.45
**VEDE**	−5.54	−5.61	−3.88	−5.58	−3.92
**NRE1**	3.47	3.57	0.49	3.52	0.40
**NRE2**	2.06	1.93	0.17	2.22	0.39
**NER3**	−3.67	−3.71	−0.41	−3.62	−0.23
**NER4**	1.86	1.82	0.04	1.89	0.23
**DM**	12.63	14.23	16.89	15.05	12.90
**Energy GAP**	9.65	9.60	9.68	9.72	9.67
**Energy GAP^ZVPE^**	9.11	9.08	9.16	9.23	9.17
****Electronic Properties****	**M06-2X/6-31+G(d,p)**				
	**TD-DFT M06-2X/6-31++G(d,p)**				
	**AQ-PO-dG**	**[*R*_P_] Slow**		**[*S*_P_] Fast**	
		**O=P-SH**	**S=P-OH**	**O=P-SH**	**S=P-OH**
**Singlet Excited Energy**	3.25	3.24	3.25	3.25	3.14
	3.52	3.51	3.39	3.51	3.41
	4.16	4.14	3.50	4.16	3.52
**Triplet Excited Energy**	2.90	2.89	2.90	2.90	2.90
	3.14	3.14	3.14	3.14	3.25
	3.23	3.27	3.26	3.27	3.28
**DM**	12.70	14.31	16.88	15.24	12.90
**GAP_LUMO/HOMO_**	5.04	4.99	5.00	5.05	5.00

### Theoretical (DFT) Study

Following the obtained experimental results, a theoretical approach was used for their clarification. Details of DFT calculation in the aqueous phase are given in the Supplementary Materials. As a model, the AQ-PO-dG and AQ-PS-dG (Figure 1) was chosen. It is important to point out here that the theoretical results obtained at the CBS-QB3 level of theory are in accordance with the experimental crystallographic/thermodynamic data of Frey, Boorman and Uhlenbeck. Based on this, the sulphur protonated form of a PT internucleotide bond was chosen [21,22,23,24]. Moreover, the proposed structures serve as a model in a correctly expressed physiological situation, where the p*K*_a_ value of the PT diester bond was found to be 2.2 lower than that of the phosphate one [21]. Therefore, the internucleotide phosphothioate bond should possess a net negative charge localised on the sulphur atom [22,23,24,25]. However, in light of the atom’s electronegativity, protonation of oxygen can be expected. Consequently, both the internucleotide bond variants O=P-SH and S=P-OH were taken into theoretical consideration (Figure 1).

Besides the phosporothioate/native diester linkage, these molecules possessed an anthraquinone moiety and the 2′-deoxyguanine (dG), dG was recognised as a nucleobase with the lowest ionisation potential [26]. Therefore, it was expected that any influence of the PT linkage on the electrochemical properties of the discussed molecules should be distinctly noticeable under a DFT study in the aqueous phase. As depicted in Figure S6, seven parameters describe the ability of a molecule to attach or detach the electron, namely: adiabatic ionisation potential (AIP), adiabatic electron affinity (AEA), vertical ionisation potential (VIP), vertical electron affinity (VEA), vertical electron detachment energy (VEDE), vertical electron affinity of cation (VEAC), and the energy difference between AIP and AEA (*i.e.*, GAP) (see Supplementary Material) [27,28,29,30]. The results of the theoretical calculations are presented in Table 2. Each DFT study was performed using Gaussian 09 [31].

In the study, the following order of adiabatic (with or without zero-point vibration energy (ZVPE) correction) and vertical ionisation potential was assigned: [*R*_P_] AQ-PS-dG < AQ-PO-dG < [*S*_P_] AQ-PS-dG for both variants discussed. The following orders of vertical electron affinity of the cation were found: [*S*_P_] AQ-PS-dG < [*R*_P_] AQ-PS-dG < AQ-PO-dG and AQ-PO-dG < [*S*_P_] AQ-PS-dG < [*R*_P_] AQ-PS-dG for the O=P-SH and S=P-OH respectively.

These data show that the configuration on the phosphorus atom in a phosphorothioate internuclotide bond can play a significant role during the charge migration through *ds*-DNA. The presence of [*R*_P_] PT linkage decreases the IP value by 0.16 eV/0.12 eV (O=P-SH/S=P-OH) in comparison to AQ-PO-dG. The IP^ZPVE^ values have been found at the same level for both variants IP^ZVPE^ = 0.14 eV. On the other hand, the appearance of [*S*_P_] PT linkage increases the IP value by 0.07 eV/0.03 eV and IP^ZVPE^ by 0.06 eV/0.12 eV for the O=P-SH and S=P-OH forms respectively. Surprisingly, a higher vertical electron attachment energy was assigned for AQ-PO-dG (see Table 2) in the case of the sulphur protonated form. The VEAC (O=P-SH) differences between AQ-PO-dG/[*R*_P_]AQ-PS-dG and AQ-PO-dG/[*S*_P_]AQ-PS-dG were found as follows: 0.03 eV and 0.10 eV respectively. The situation was found to be the opposite for the oxygen protonated variant, with the AQ-PO-dG exhibiting a lower value than phosphorothioate derivatives *R*_P_ and *S*_P_. The following differences were found: 1.77 eV (AQ-PO-dG/[*R*_P_]AQ-PS-dG) and 1.70 eV (AQ-PO-dG/[*S*_P_]AQ-PS-dG). Finally the nuclear relaxation energies were examined, and it was found that the [*R*_P_] AQ-PS-dG demanded a lower energetic effort for both the discussed O=P-SH and S=P-OH variants (Table 2). Based on the ionisation potential values and on the sum of the nuclear relaxation energy (NRE1 and 2, Table 2), it can be concluded that in both protonated forms, [*R*_P_] AQ-PS-dG demonstrates a higher ability to form and stabilise the cation radical among all the discussed molecules. However, derivatives in which the oxygen atom is protonated showed better electronic properties towards the stabilisation of the cation radical than those found for the O=P-SH variant (Table 2).

From another point of view, the attachment of an electron by a molecule leads to a radical anion formation. For stable anionic forms, EA adopts a positive value. Conversely, a negative EA indicates anion instability and a significantly shorter lifetime. Initially, the attachment of an excess electron leads to “transient negative ion” formation without spatial geometry reorganization [32]. Therefore, in this state, the vertical attachment energy determines the energy necessary for fast electron capture by the molecule. For all the investigated molecules the vertical electron affinity energies adopted positive values (see Table 2), in the following order: [*R*_P_] AQ-PS-dG > [*S*_P_] AQ-PS-dG > AQ-PO-dG for the sulphur protonated forms and [*R*_P_] AQ-PS-dG ~ [*S*_P_] AQ-PS-dG > AQ-PO-dG for the oxygen protonated forms. These data indicate that the presence of the PT internucleotide bond, depending on the configuration on the phosphorus atom [*R*_P_] or [*S*_P_], is substantial for initially attracting the electron; if the sulphur atom is protonated, the difference is negligible. In the case of the oxygen protonated form, the *R*_P_ and *S*_P_ forms of AQ-PS-dG adopt almost identical VEA values: 3.43 eV and 3.45 eV, respectively. The electron attracted in the initial stage was sequentially delocalized over [*R*_P_] AQ-PS-dG, [*S*_P_] AQ-PS-dG, and AQ-PO-dG. The dispersion of negative charge led to changes in geometry, manifested as relaxation energy corresponding to the adiabatic electron affinity. All the discussed molecules showed a positive value of AEA and AEA^ZPVE^ energies (in eV) in the following order: [*R*_P_] AQ-PS-dG > [*S*_P_] AQ-PS-dG ~ AQ-PO-dG (Table 2). This was independent of sulphur or oxygen protonation in the PT bond, which indicates that radical anionic forms are allowed for the discussed conjugates. Moreover, the phosphorothioate [*R*_P_] showed a higher electron affinity and VEDE (Table 2) in both the O=P-SH and S=P-OH variants. However, for the above-mentioned molecule, the relaxation energy, after electron detachment *i.e.*, NRE 4 (O=P-SH), adopted a lower value: 1.816 eV, *vs.* 1.888 eV and 1.856 eV for [*S*_P_] AQ-PS-dG and AQ-PO-dG respectively. In the case of S=P-OH the following VEDE values were found in eV: 0.04 [*R*_P_] AQ-PS-dG, 0.23 [*S*_P_] AQ-PS-dG (Table 2).

Based on these results it can be tentatively predicted that [*R*_P_] AQ-PS-dG should exhibit some predisposition to excess electron attachment in both protonated forms of the PT bond. The difference between ZVPE aorected/uncorected AIP and AEA, *i.e.*, GAP, indicates the stability of the radical forms formed by molecules. The lower value of GAP indicates the more stable radical. The GAP/GAP^ZPEV^ energy was found to range between: 9.60 eV/9.08 eV and 9.71 eV/9.23 eV. Based on this relationship and the obtained results presented in Table 2, it can be concluded that the radicals formed by each of the discussed molecules (S=P-OH and O=P-SH variants) should show the same stability.

It is important to mention here that a comparison of the electronic properties of both diastereomers *R*_P_ and *S*_P_ of AQ-PS-dG in the different protonated forms of the internucleotide phosphorothioate bond did not show a significant influence on the AIP, AEA and GAP energy. However, from the energetic point of view, the protonation of the oxygen atom was found to be favoured. For all the molecules, in their optimized geometries, the energies (E° and E^ZVPE^) for the S=P-OH variant were found to be low in comparison with those of O=P-SH, as shown in Table S4.

Therefore, the presence of [*R*_P_] phosphorothioate (with a lower AIP value) should protect the oligonuncleotide against lesion formation by hindering the charge transfer through *ds*-DNA independently of sulphur or oxygen protonation. This is most probably why *oligo*-B showed a higher stability under UVA irradiation conditions than native *ds*-DNA *oligo*-A (see Figure 2).

Additionally, for both investigated forms O=P-SH and S=P-OH, the TD-DFT [33] energy calculation elucidated that the [*R*_P_] AQ-PS-dG adopts preferably a triplet excited state (Table 2). Moreover, for all the discussed molecules, the formation of a triplet excited state was found to be preferable to the singlet. These results correspond well with previous data [13]. Additionally, the calculated GAP_HOMO/LUMO_ energy, based on HOMO and LUMO energies [28], which can be roughly recognized as IP and EA energies according to Koopmans’ theorem, confirm the ability of [*R*_P_] AQ-PS-dG to form a stable radical independently of sulphur or oxygen atom protonation in the phosphordiester bond.

## 3. Experimental Section

The experimental section has been attached as Electronic Supplementary Files which contain: the synthesis and characterization of oligonucleotides, CD spectra, Tm value, MALDI TOF. Characterization of AQ-PO-dG and AQ-PS-dG molecules: UV, MALDI TOF, RP HPLC analysis. Preparation of radiolabeled oligonucleotides, procedure of irradiation and cleavage analysis.

## 4. Conclusions

Based on the experimental findings presented above, the following conclusions can be proposed:

(1) the distal/terminal PT internucleotide bond present in *ds*-DNA significantly slowed down the charge migration;
(2) probably the presence of sulphur in the diester bond (phosphorothioate), which covalently links AQ to the oligonucleotide, reduced the oxidation process due to the disturbance of a mutual AQ/oligonucleotide spatial interaction;
(3) a comparison of the electronic properties of both diastereomers *R*_P_ and *S*_P_ of AQ-PS-dG did not show a significant influence of the different protonated forms of the PT bond on AIP, AEA and GAP energies, or on the ZPVE corrected variants, either. Therefore, the influence of oxygen or sulphur protonation of PT bond on the “hole” migration process should lead to barely perceptible changes;
(4) the theoretical study showed that [*R*_P_] AQ-PS-dG can adopt a lower energy of ionisation potential and triplet excited state with a subsequent higher electron affinity value. Moreover, the energy gap between HOMO and LUMO, calculated by TD-DFT methodology, indicated the radical stabilisation properties of [*R*_P_] AQ-PS-dG, which can hinder the undisturbed charge transfer through *ds*-DNA.

## Supplementary Materials

Supplementary materials can be accessed at: http://www.mdpi.com/1420-3049/20/07/12400/s1.
