# Supplementary material for: The Influence of the Terminal Phosphorothioate Diester Bond on the DNA Oxidation Process. An Experimental and Theoretical Approach
Source: Molecules. 2015 Jul 8;20(7):12400–11. doi: 10.3390/molecules200712400 (PMC6331877; doi:10.3390/molecules200712400)
Supplement: Supplementary file 1 [file molecules-20-12400-s001.pdf]

# Supplementary Information

## S1. Materials and Methods

### *S1.1. Synthesis and Purification*

Oligonucleotides, sequence given below, were synthesized in the Bioorganic Chemistry Department, Polish Academy of Science, Lodz, Poland on an on an Geneworld (K & A Laborgeraete GbR, Schaafheim, Germany) synthesizer and using nucleotide phosphoroamidites purchased from ChemGenes Corporation. The phosphoroamidite derivative of anthraquinone was synthetized as described previously [1].

The crude oligonucleotides were purified by HPLC using: Varian analyticsystem with UV detection in dual wavelengths 260 and 334 nm, Phenomenex (Synergi 4u Fusion-RP 80A, 250 × 4.6 mm) C-18 column. The elution was achieved using a 0.1 M ammonium acetate solution in water as a buffer "A" at pH-7, with a gradient from 0% to 40% of buffer "B" (40% acetonitrile in buffer "A") over 40 min, then from 40% to 0% of buffer "B" over 5 min, followed by 0% of buffer "B" over 5 min.

### *S1.2. Mass Spectroscopy*

The obtained oligonucleotides were characterised by mass spectroscopy MALDI TOF. Negative ion MALDI mass spectra were recorded on a Voyager-Elite (PerSeptiveBiosystems Inc., Framingham, MA, USA) instrument equipped with nitrogen laser (337 nm) in a linear mode at an acceleration voltage of 20 kV and delayed extraction.

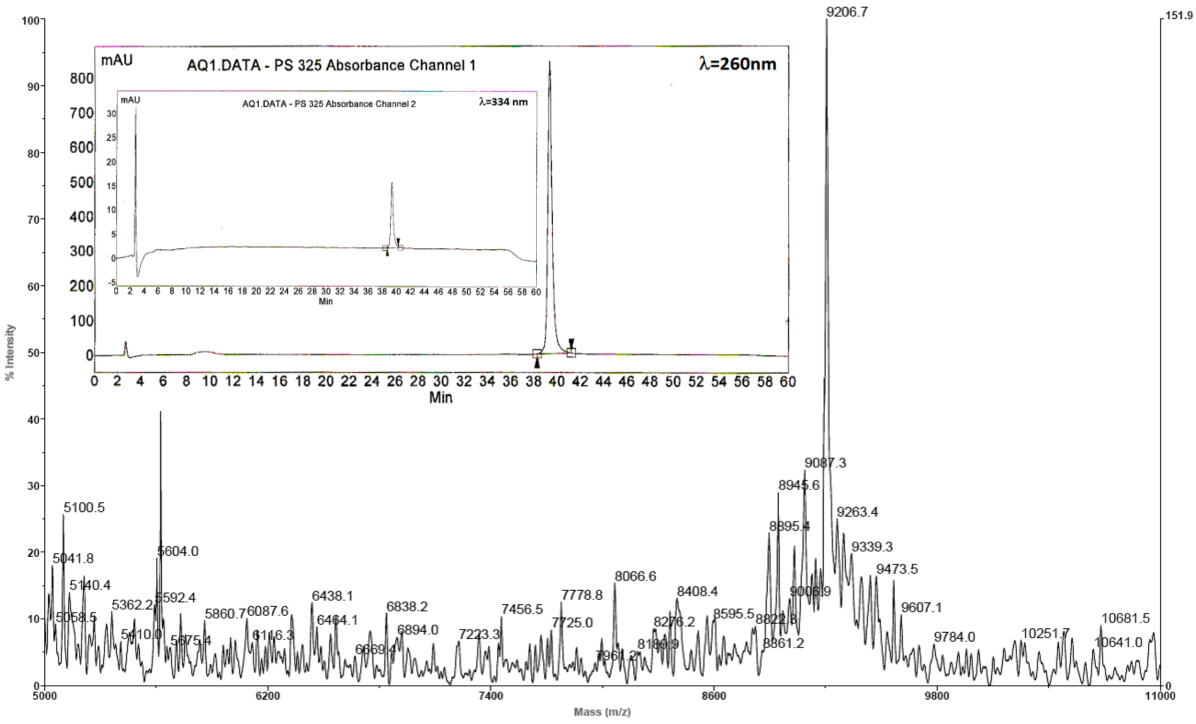


**Figure S1.** MALD TOF spectra and RP-HPLC profile of **AQ-PO-29**.

**Table S1.** Sequence and MALDI TOF analysis of investigated oligonucleotides.

| | Oligonucleotide Sequence | Calculated Mass | Obtained Mass |
|---|---|---|---|
| **A** | 5′-AAATTAATATGTATTGTATATAAATTATT-3′ | 8927 | 8927 |
| **AQ-PO-29** | 3′-TTTAATTATACATAACATATATTTAATAA-**PO**-AQ-5′ | 9215 | 9207 |
| **AQ-PS-29** | 3′-TTTAATTATACATAACATATATTTAATAA-**PS**-AQ-5′ | 9231 | 9223 |

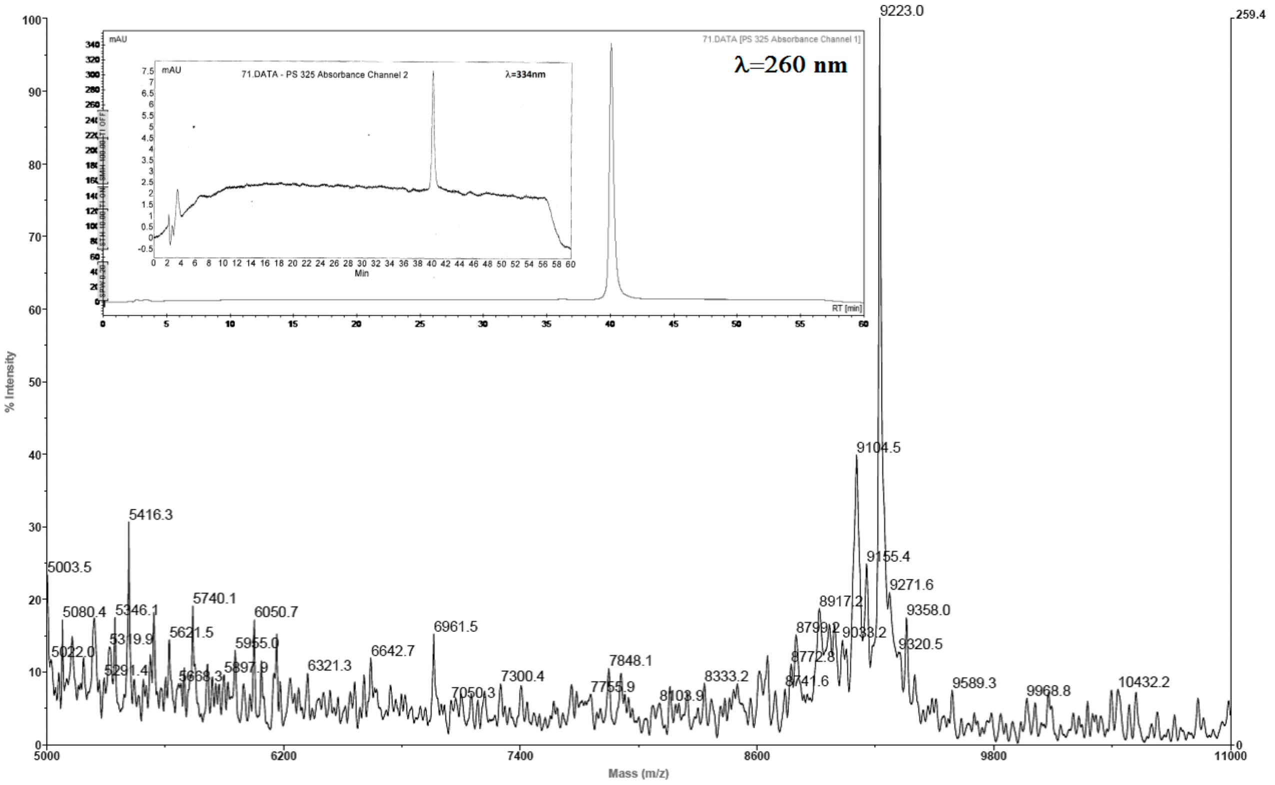


**Figure S2.** MALD TOF spectra of **AQ-PS-29** and RP-HPLC profile.

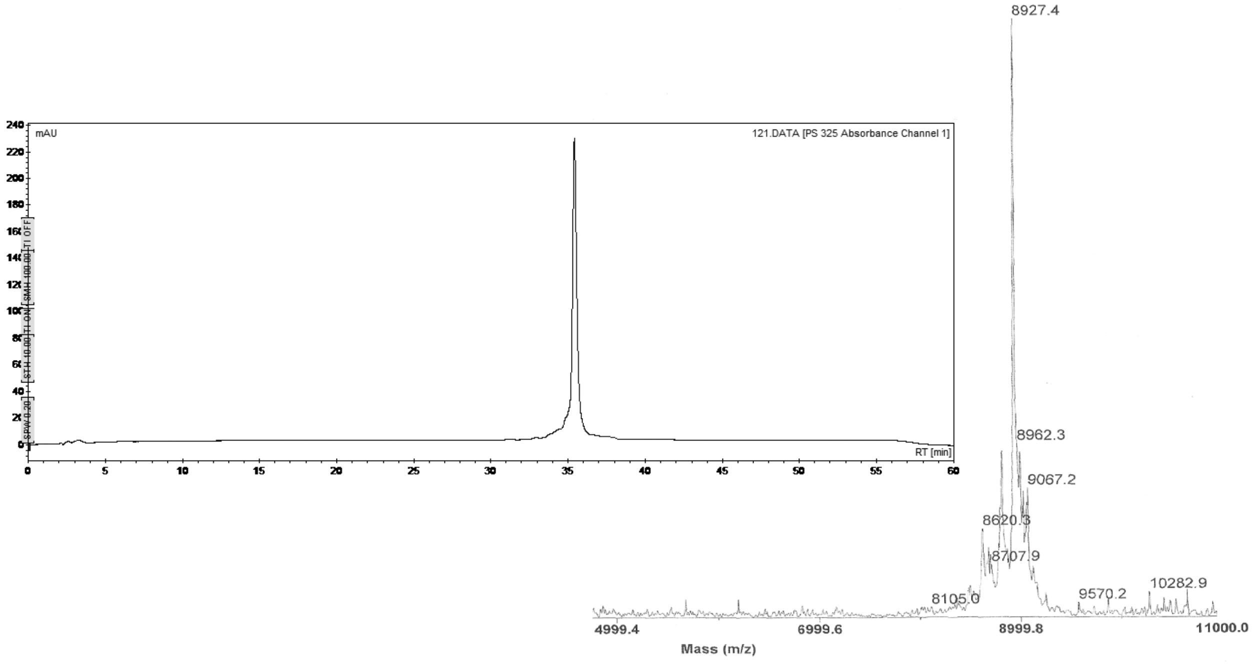


**Figure S3.** MALD TOF spectra of oligodeoxynucleotide **A** and RP-HPLC profile at 260 nm.

*S1.3. Concentration*

The concentration of obtained oligonucleotides were determinated from a maximum of absorbance ~260 nm using a Hitachi U-2800 spectrophotometer. The online oligonucleotide properties calculator (OligoCalc) was used for the extinction coefficient determination of oligonucleotides in the case of AQ-PO-29, and AQ-PS-29 adenine was used instead of antraquinon moiety [1].

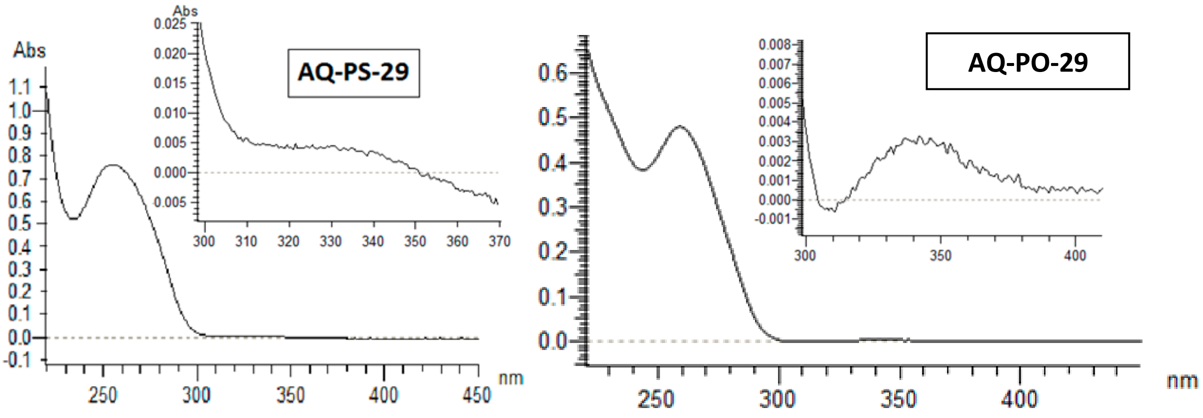


**Figure S4.** UV spectra of AQ-PS-29 and AQ-PO-29 oligonucleotides.

*S1.4. Melting Temperature*

The melting temperatures ($T_m$) were assigned on a Cary 1.3E spectrophotometer equipped with a multicells block and temperature controller. The required amount of each oligonucleotide strand (2.9 µM) was dissolved in 1 mL of 0.1 M NaCl with 0.01 $MgCl_2$ and hybridized by heating at 90 °C for 10 min followed by slow cooling (overnight). Melting profiles were acquired by measuring the absorbance at a wavelength of 260 nm as a function of temperature. The values, presented in table $T_m$, were measured in a range between 20 and 80 °C with 0.5 °C/min step and 1 min of holding time.

**Table S2.** Melting temperatures, as a maximum of first derivatives, obtained for double-stranded oligonucleotides, *ds*-oligonucleotides sequences given in Table 1 in the main text of article.

| ***ds*-DNA** | **T*m* [°C]** | |
|---|---|---|
| | **AQ(+) *** | **AQ(−)** |
| ***oligo*-A** | 58.64 | 53.33 |
| **oligo-A **** | 57.32 | |
| ***oligo*-B** | 52.70 | 53.33 |
| **oligo-B **** | 51.43 | |

***AQ(+)** complementary strand contains anthraquinone unit (AQ-PO-29, AQ-PS-29); **AQ(−)** complementary strand without anthraquinone unit (oligonucleotide F); ** $T_m$ value obtained after UV irradiation: 350 nm, 120 min.

*S1.5. Circular Dichroism Analysis*

CD spectra were recorded with a CD6 dichrograph (Instruments SA JobinYvon, Longjumeau, France) at room temperature using 0.1 cm quartz cuvettes and an oligonucleotide concentration of 1.32–1.5 μM, as this concentration permitted parallel UV measurements. The CD spectra were collected within the range of 200 to 320 nm with an integration time of 1 s, increment 1 nm, bandpass 2 nm. The profiles of the obtained spectra are given in below.

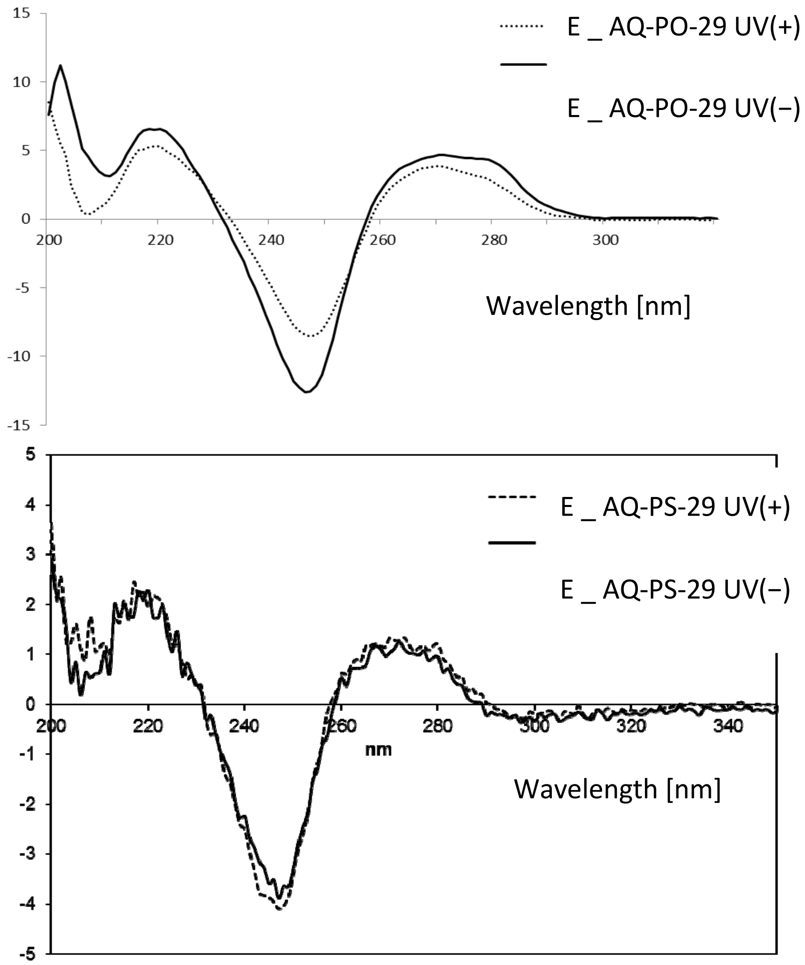


**Figure S5.** Circular dichroism spectra of investigated oligonucleotides. UV (+) indicated that the sample was previously irradiated by 350 nm light for 120 min, UV (−) dark sample.

*S1.6. Preparation of 5′-End-Labeled Oligonucleotides*

The oligonucleotides (0.06 μM) were 5′-End-labeled using 3.2 unit of T4 polynucleotide kinase (New England BioLabs, Ipswich, MA, USA) with 1.6 mCi(1.6 μL) [γ-$^{32}$P]ATP (PerkinElmer, Warsaw, Poland) in 16 μL of buffer [70 mM Tris-HCl (pH 7.6), 10 mM $MgCl_2$, 100 mM KCl, and 1 mM β-2-mercaptoethanol] for 30 min at 37 °C. After incubation, the radiolabeled sample was filtrated through a MicroSpin G-25 column (GE Healthcare, Buckinghamshire, UK), at 6000 RPM/2 min, the residue evaporated to dryness. The purity of the oligonucleotides investigated were examined on a 20% denaturing poliarylamide gel.

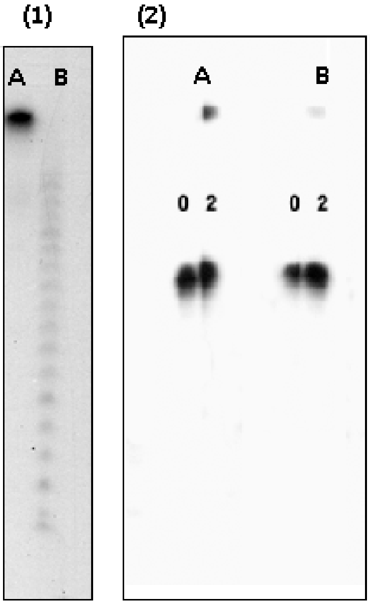


**Figure S6. (1)** Autoradiogram showing the purity of the investigated oligonucleotide. (Denaturing 7M urea 20% poliarylamide gel electrophoresis) Line A: oigonucleotide A with sequence given in Table S1, B: Crude product of AQ-PS-29 synthesis; **(2)** Autoradiogram of irradiation *ds*-DNA after: (0) 0 min and (2) 120 min without piperidine cleavage. (Denaturing 7M urea 20% poliarylamide gel electrophoresis) Line A *oligo*-A and B *oligo*-B, are corresponding to double-stranded oligonucleotides sequences given in Table 1, main text of article.

*S1.7. Hybridisation, UVA Duplex Irradiation and Cleavage Analysis*

The labelled oligonucleotide was hybridized, as described previously, with a 2-fold excess of the purified non-radiolabelled complementary strand in 100 μL of 0.1 M NaCl with a 0.01 M $MgCl_2$ buffer solution at pH 6.7. After hybridization, the total amount of oligonucleotides solution was divided into 10 μL samples and irradiated for 0, 30, 60 and 120 min at ~35 °C in a Rayonet Photoreactor RMR-600 (Southern New England Ultraviolet Co., Bradford, CT, USA) equipped with two 4W, 350 nm lamps. The procedure described previously [2] was followed: after each time or irradiation, the oligonucleotide was precipitated with cold ethanol (100 μL) and 2 μL of glycogen vortex and placed on dry ice for 30 min with subsequent centrifuged 12,000 rpm for 30 min in 4 °C. The ethanol was removed and the residue then dried under air conditioning at room temperature. To reveal the DNA lesions the dry samples of each investigated oligonucleotide was treated by 100 μL of 1 M piperidine solution at 90 °C for 30 min. The piperidine was removed by evaporation under reduced pressure. The residue of the oligonucleotides under investigation were dissolved in 7 μL of denaturing loading dye (98% formamide, 2 mM EDTA, 0.025% bromophenol blue, and 0.025% xylene cyanol). The samples were then subjected to electrophoresis on a 20% denaturing polyacrylamide gel containing 7 M urea in 1X TBE [89 mM Tris-HCl, 89 mM boric acid, and 2 mM EDTA (pH 8.3)] for 180 min at a constant power of 24 W. The results of PAGE electrophoresis analysis were visualized by autoradiography. The Quantity One 1-D analysis software (Bio-Rad) was used to estimate the cleavage bands (Figure 3).

**Table S3.** Densitometry values calculated on the band intensity shown in Figure 2 in the main text of article.

| Irradiation Time | Double Stranded Oligonucleotides | |
|---|---|---|
| | *oligo*-A | *oligo*-B |
| **120 min** | 0.381469 | 0.671314 |
| **−4 (T26)** | 0.14037 | 0.05713 |
| **9 (T21)** | 0.227552 | 0.177583 |
| **−11 (T19)** | 0.157571 | 0.069057 |
| **−14 G19)** | 0.049206 | 0.024964 |
| **60 min** | 0.625143 | 0.91118 |
| −4 | 0.129326 | 0.004026 |
| −9 | 0.141363 | 0.06519 |
| −11 | 0.075587 | 0.014622 |
| −14 | 0.028581 | 0.004981 |
| **30 min** | 0.79403 | 0.955282 |
| −4 | 0.077046 | 0.003066 |
| −9 | 0.080864 | 0.037955 |
| −11 | 0.03187 | 0.004026 |
| −14 | 0.016189 | −0.00033 |
| **0 min** | 1 | 1 |
| −4 | 0 | 0 |
| −9 | 0 | 0 |
| −11 | 0 | 0 |
| −14 | 0 | 0 |

*S1.8. Synthesis of Model System AQ-PO-dG and AQ-PS-dG*

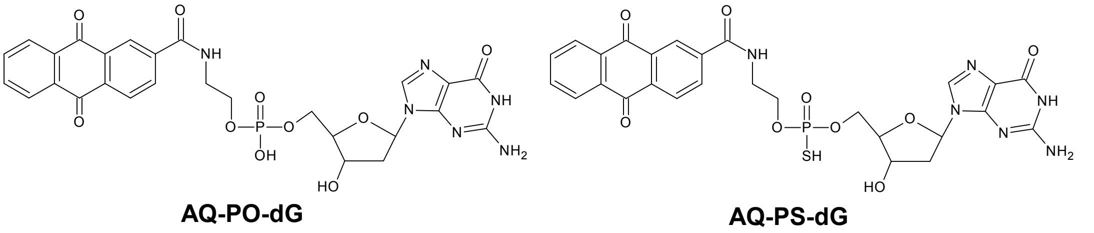


**Figure S7.** Graphical representation of AQ-PO-dG and AQ-PS-dG structures.

5′-O-DMT-N[4]-izobutyrylo-2′-deoxygyanosine (1 μmol) bound to the standard LCA CPG solid support (Glen Research, Virginia) was detritylated with a 3% solution of DCA in methylene chloride, then washed thoroughly with 10 mL of dry acetonitrile and 10 mL of dry methylene chloride, and dried. For the coupling step, a dry acetonitrile solution of *N*-(2-(methoxy(diisopropylamino) phosphinyl) ethyl)-2-anthraquinonecarboxamide (296.09) 30 mg, 300 mL) and 1-*H*-tetrazol (15 mg, 150 mL) was prepared and instantly introduced into the column. After 10 min the column was washed with *dry* acetonitrile (10 mL), 10 mL of dry methylene chloride and dried. Depending of the requirements, the transition product PIII was oxidized to AQ-PO-dG by $I_2$/Pirydine/$H_2O$ (1 mL, 5 min) or sulphurated to AQ-PS-dG by 2% S-tetra pyridine/acetohitryle 1:1 solution (1 mL, 5 min). Finally the column was washed again, and the “dinucleotide” was released from the support by treatment with concentrated

ammonia (1 mL) for 1 h. The solution was kept for another 24 h at room temperature to remove the protective group from the guanidine. Finally the ammonium was evaporated to dryness, and the residue was dissolved in a 0.1 M ammonium acetate buffer (1 mL). RP-HPLC analysis of the crude product showed the presence of AQ-PO-dG (98%) or AQ-PS-dG (80%) with a 50/50 composition of Fast to Slow forms.

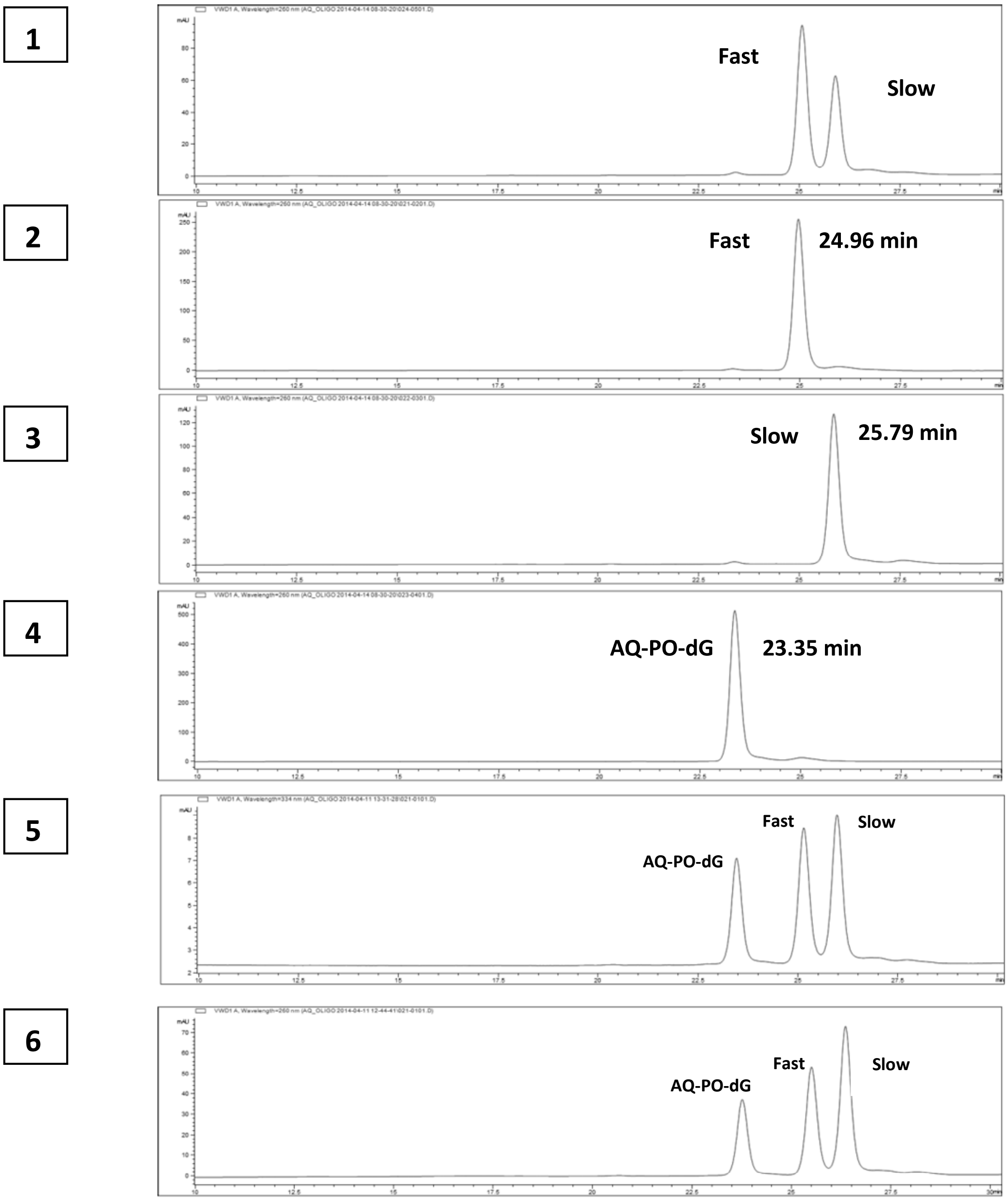


**Figure S8.** RP-HPLC Chromatograms Profiles of Synthesis Products after Purification. (**1**) RP-HPLC analysis of mixture of "Fast" and "Slow" form of AQ-PS-dG; (**2**) RP-HPLC analysis of mixture of "*Fast*" AQ-PS-dG form; (**3**) RP-HPLC analysis of mixture of "*Slow*" AQ-PS-dG form; (**4**) RP-HPLC analysis of mixture of AQ-PO-dG form; (**5**) RP-HPLC (260 nm) analysis of mixture of "Fast" and "Slow" form of AQ-PS-dG and AQ-PO-dG; (**6**) RP-HPLC (334 nm) analysis of mixture of "Fast" and "Slow" forms of AQ-PS-dG and AQ-PO-dG.

The crude products were purified by HPLC using the following: Varian analytical system with UV detection in dual wavelengths 260 and 334 nm, Phenomenex (Synergi 4u Fusion-RP 80A, 250 × 4, 6 mm) C-18 column. The elution was achieved using a 0.1 M ammonium acetate solution in water as a buffer “A” at pH-7, with a gradient from 0% to 40% of buffer “B” (acetonitrile HPLC grade) over 40 min, then from 40% to 0% of buffer “B” over 5 min, followed by 0% of buffer “B” over 5 min.

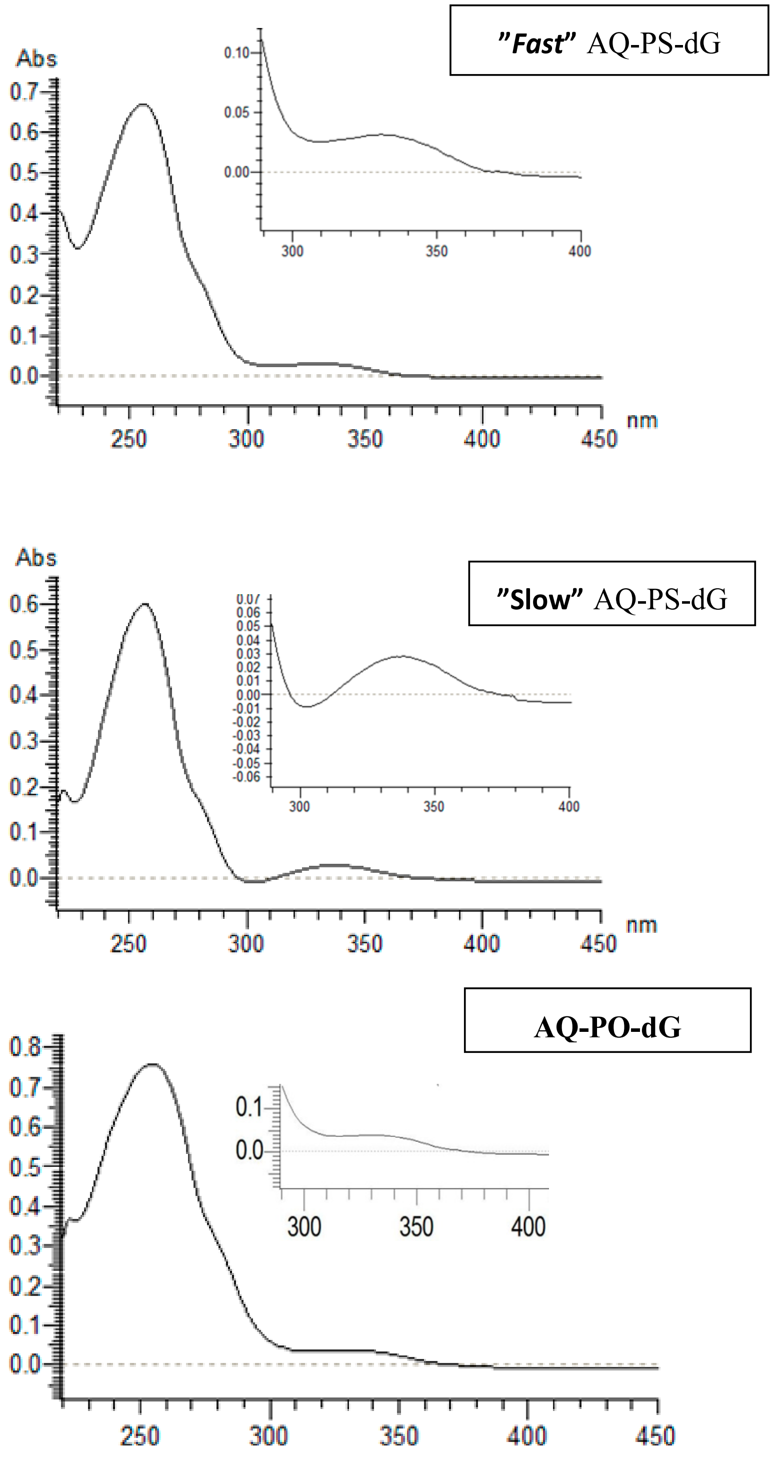


**Figure S9.** UV Spectra of AQ-PO-dG, “Fast” and “Slow” AQ-PS-dG Dissolved in 0.1 M Ammonium Acetate.

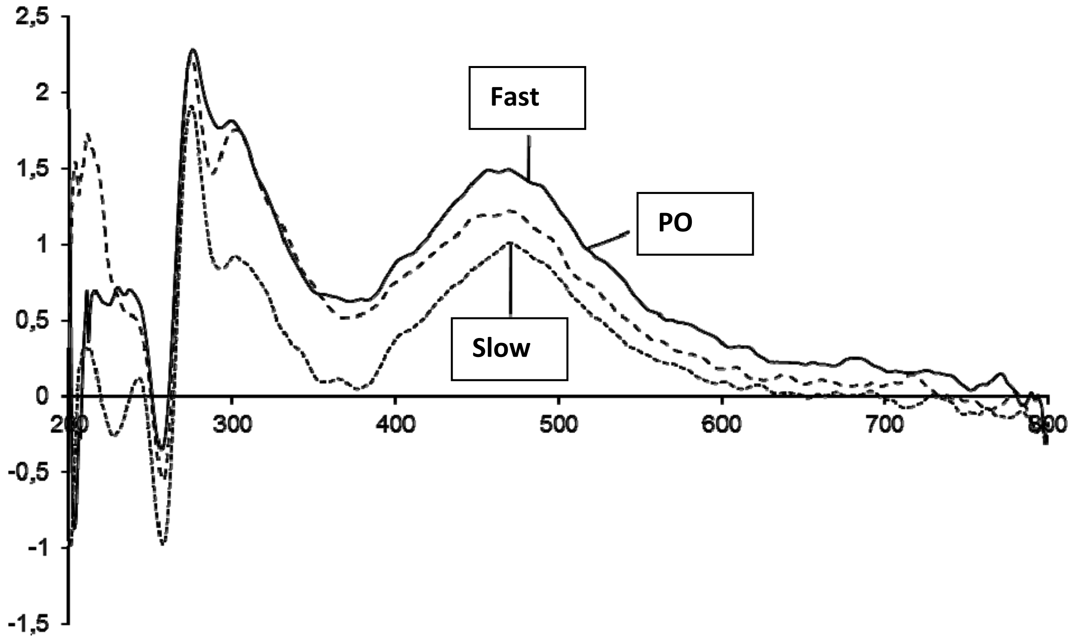


**Figure S10.** CD crude spectra without buffer baseline subtraction of PO (*i.e.*, AQ-PO-dG), "*Fast*" and "*Slow*" forms of AQ-PS-dG dissolved in 0.1 M ammonium acetate.

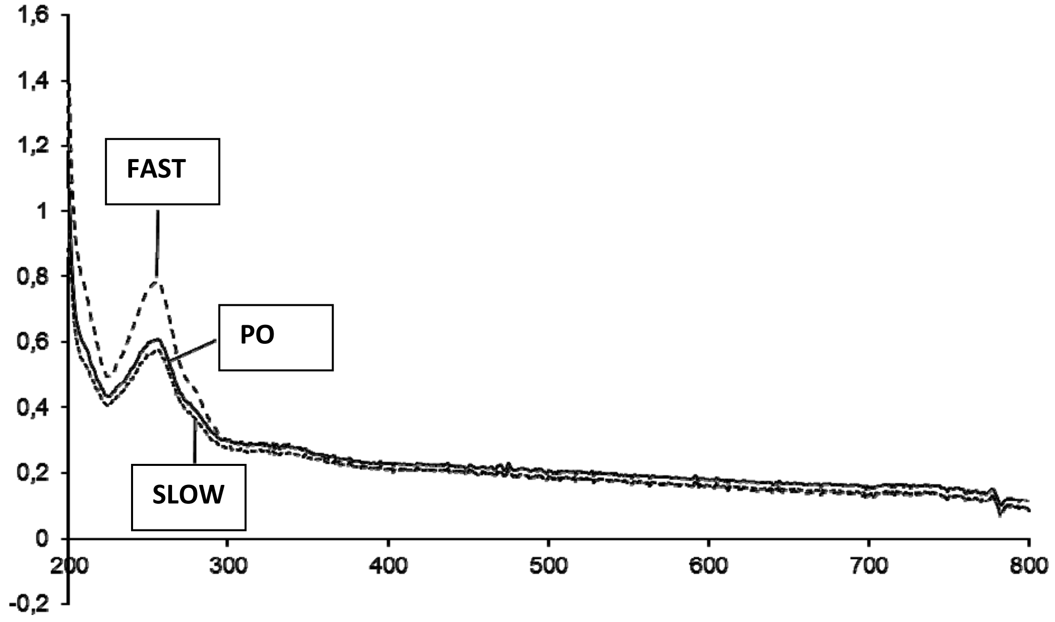


**Figure S11.** CD spectra of used buffer (baseline correction): AQ-PO-dG, "*Fast*" and "*Slow*" forms of AQ-PS-dG dissolved in 0.1 M ammonium acetate.

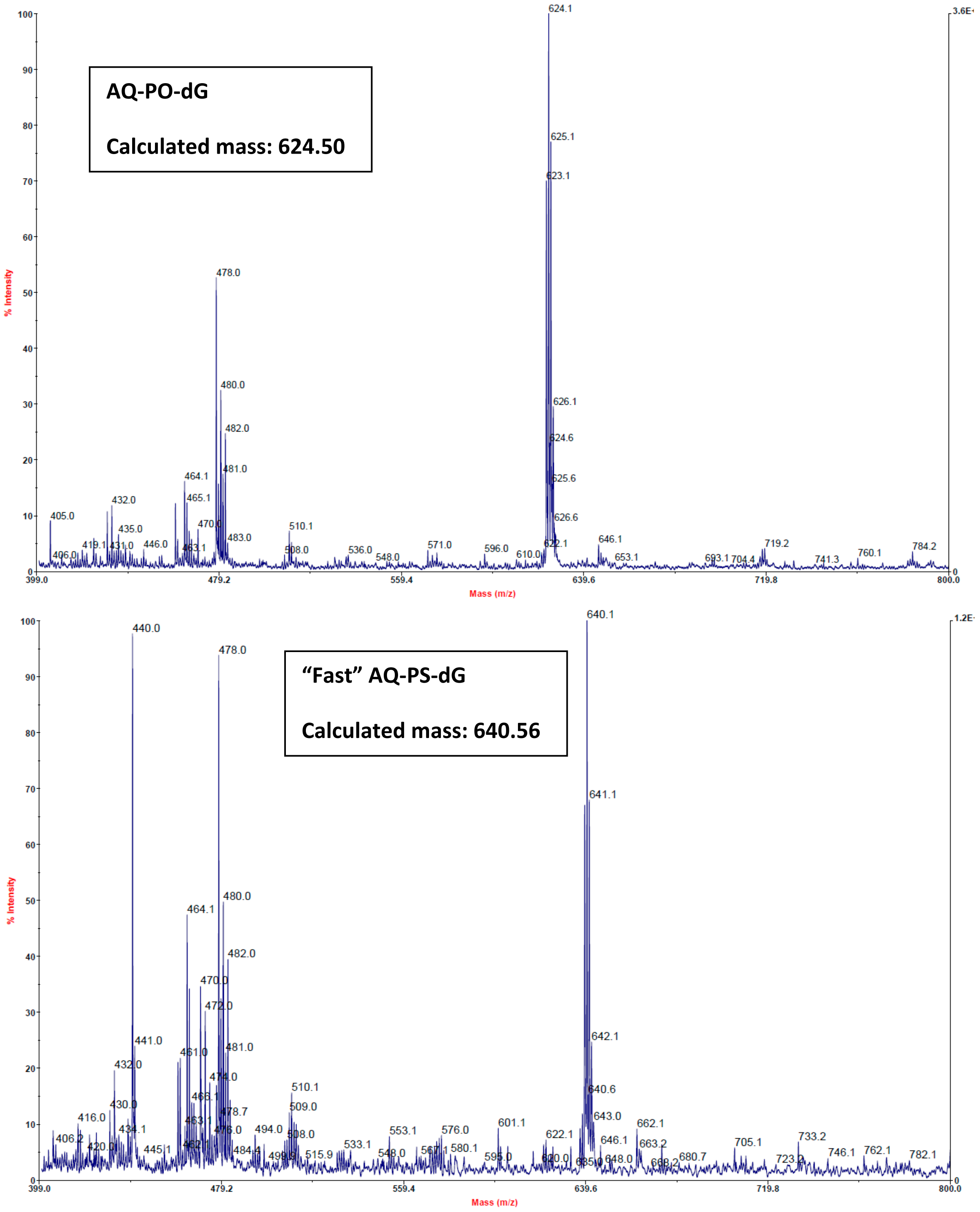


**Figure S12.** *Cont.*

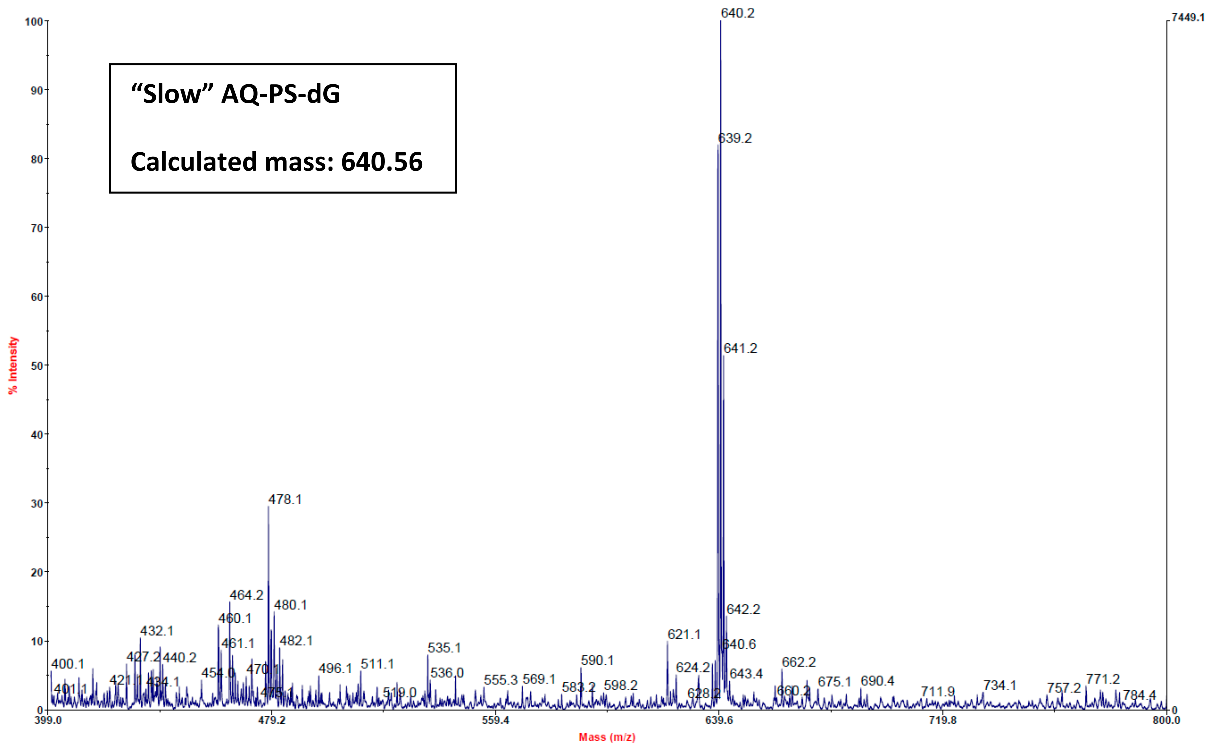


**Figure S12.** Mass Spectroscopy analysis (MALDI TOF) of AQ-PO-dG, ”*Fast*” and “*Slow*” forms of AQ-PS-dG.

Computation Methodology of Quantum Mechanics Study

All the geometries optimization calculations were performed by the density functional theory (DFT) using the M06-2X functional [3,4]. M06-2X is a hybrid meta-generalized gradient approximation (GGA) exchange functional (DFT method) developed by Truhlar, who recommended augmented polarized valence double-ζ basis set 6-31+G(d,p) for this functional [5]. For characterisation of the stationary point (no imaginary frequencies) of all the investigated molecules, the harmonic vibration was calculated at the M06-2x/6-31G** level of theory. Moreover, using this strategy, the contribution of zero-point vibrational correction and thermal contribution to the free energies was considered. For the characterisation of the singlet and triplet excited states the single point calculation at the M06-2X/6-31++G(d,p) level of theory were performed using the time-dependent DFT (TD-DFT) methodology [6]. The appropriate adiabatic electron affinities (AEA) were obtained as the difference between the energies of the appropriate neutral form ($E_{Neutral}$) and anion one ($E_{Anion}$) at their optimized geometries **AEA** = $\boldsymbol{E_{Neutral}(r_e,0) - E_{Anion}(r_e,^-)}$ [7]. In this study, the electron affinity is defined as the released energy when the electron is added to a neutral molecule [8].

The vertical electron affinities (**VEA**) were obtained as the difference between the energies of the appropriate neutral form ($E_{Neutral}$) and anion one ($E_{Anion}$) at optimized neutral geometries **VEA** = $\boldsymbol{E_{Neutral}(r_e,0) - E_{Anion}(r_e,0)}$ [7].

The vertical electron detachment energy (**VEDE**) were obtained as the difference between the energies of the appropriate neutral form ($E_{Neutral}$) and anion one ($E_{Anion}$) at optimized anion geometries **VEDE** = $\boldsymbol{E_{Anion}(r_e,^-) - E_{Neutral}(r_e,^-)}$ [9].

From the definition, the ionisation potential is the amount of energy required to remove an electron from molecule. The suitable adiabatic ionisation potential (AIP) was obtained as the difference between the energies of the appropriate cationic form ($E_{Cation}$) and neutral one ($E_{Neutral}$) at their optimized geometries **AIP** = $\boldsymbol{E_{Cation}(r_e,^{+}) - E_{neutral}(r_e,0)}$ [10].

The vertical ionization potential VIP were obtained as the difference between the energies of the appropriate cation form ($E_{Cation}$) and neutral ($E_{Neutral}$) one at optimized neutral geometries **VIP** = $\boldsymbol{E_{Cation}(r_e,0) - E_{Neutral}(r_e,0)}$ [10].

The vertical electron affinity of cation (VEAC) were obtained as the difference between the energies of the appropriate neutral form ($E_{Neutral}$) and cation one ($E_{Cation}$) at optimized cation geometries **VEAE** = $\boldsymbol{E_{Cation}(r_e,^{+}) - E_{Neutral}(r_e,^{+})}$ [9].

All calculations performed in aqueous phase on Gaussian 09 revision A.02 software package [11].

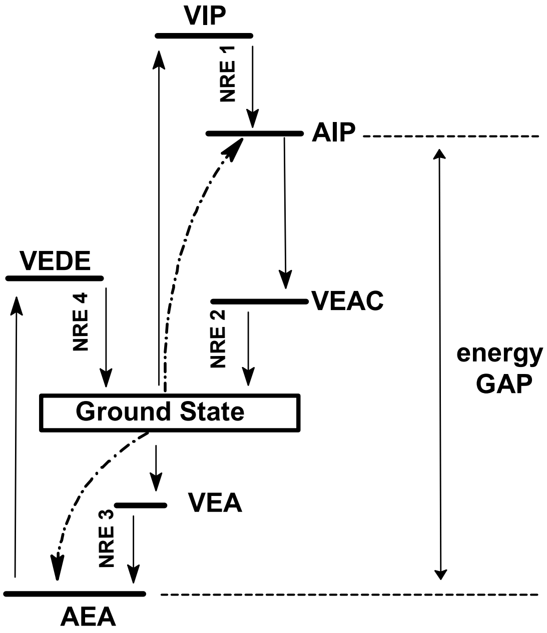


**Figure S13.** The schematic graphical representation of energy changes during the electron attachment/detachment to/from the AQ-PO-dG and AQ-PS-dG systems.

**Table S4.** Energies, in Hartree, of AQ-PO-dG, [*R*P] AQ-PS-dG, [*S*P] AQ-PS-dG calculated at M06-2X/6-31+G(d,p) level of theory, in aqueous phase.

| Molecule Form | Energies in Hartree | | | | |
|---|---|---|---|---|---|
| | AQ-PO-dG | [*R*P] AQ-PS-dG | | [*S*P] AQ-PS-dG | |
| | O=P-OH | O=P-SH | S=P-OH | O=P-SH | S=P-OH |
| | M06-2X/6-31+G** | | | | |
| Neutral $(r_e,0)$ | −2465.492835 | −2788.421887 | −2788.434997 | −2788.42414 | −2788.438010 |
| Neutral $(r_e,^{-})$ | −2465.424636 | −2788.355146 | −2788.561032 | −2788.354761 | −2788.564903 |
| Neutral $(r_e,^{+})$ | −2465.417115 | −2788.351076 | −2788.202254 | −2788.342518 | −2788.203293 |
| Cation $(r_e,^{+})$ | −2465.273697 | −2788.208555 | −2788.220376 | −2788.20259 | −2788.217955 |
| Cation $(r_e,0)$ | −2465.146137 | −2788.077269 | −2788.428840 | −2788.073133 | −2788.423692 |
| Anion $(r_e,^{-})$ | −2465.628154 | −2788.561172 | −2788.573403 | −2788.559683 | −2788.573403 |
| Anion $(r_e,0)$ | −2465.493388 | −2788.424925 | −2788.433597 | −2788.426637 | −2788.429398 |

**Table S4.** *Cont.*

| Molecule Form | Energies in Hartree | | | | |
|---|---|---|---|---|---|
| | AQ-PO-dG | [$R_P$] AQ-PS-dG | | [$S_P$] AQ-PS-dG | |
| | O=P-OH | O=P-SH | S=P-OH | O=P-SH | S=P-OH |
| | M06-2X/6-31G** (**a**-$E^0$; **b**-$E^{ZPVE}$) | | | | |
| Neutral ($r_e$,0) | a) −2465.417440 | −2788.347374 | −2788.361782 | −2788.349487 | −2788.366162 |
| | b) −2464.827441 | −2787.761941 | −2787.774231 | −2787.764567 | −2787.777717 |
| Cation ($r_e$,$^+$) | −2465.206841 | −2788.142555 | −2788.157572 | −2788.136325 | −2788.154128 |
| | −2464.617137 | −2787.556977 | −2787.569197 | −2787.549946 | −2787.565411 |
| Anion ($r_e$,$^-$) | −2465.54045 | −2788.47274 | −2788.491142 | −2788.472151 | −2788.488511 |
| | −2464.95194 | −2787.890656 | −2787.90582 | −2787.888951 | −2787.902209 |
| | 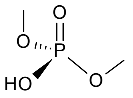 | 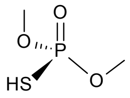 | 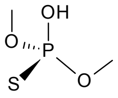 | 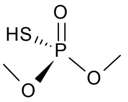 | 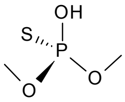 |

## References


1. Gasper, M.S.; Shuster, G.B. Intramolecular Photoinduced Electron Transfer to Anthraquinones Linked to Duplex DNA: The Effect of Gaps and Traps on Long-Range Radical Cation Migration. *J. Am. Chem. Soc.* **1997**, *119*, 12762–12771.
2. Ghosh. A.; Joy, A.; Schuster, G.B.; Duki, T.; Cadet, J. Selective one-electron oxidation of duplex DNA oligomers: reaction at thymines. *Org. Biomol. Chem.* **2008**, *6*, 916–928.
3. Zhao, Y.; Pu, J.; Lynch, B.J.; Truhlar, D.G. Tests of second-generation and third-generation density functionals for thermochemical kinetics. *PCCP* **2004**, *6*, 673–676.
4. Zhao, Y.; Truhlar, D.G. Design of Density Functionals That Are Broadly Accurate for Thermochemistry, Thermochemical Kinetics, and Nonbonded Interactions. *J. Phys. Chem. A* **2005**, *109*, 5656–5667.
5. Hehre, W.J.; Radom, L.; Schleyer, P.; Pople, R.J.A. *Ab Initio Molecular Orbital* Theory; Wiley: New York, NY, USA, 1986; pp. 63–101.
6. Rugne, E.; Gross, E.K.U. Density-Functional Theory for Time-Dependent Systems. *Phys. Rev. Lett.* **1984**, *52*, 997–1000.
7. Rienstra-Kiracofe, J.C.; Tschumper, G.S.; Schaefer, H.F., III; Nandi, S.; Ellison, B. Atomic and Molecular Electron Affinities: Photoelectron Experiments and Theoretical Computations. *Chem. Rev.* **2002**, *102*, 231–282.
8. Zhan, C.-G.; Nichols, J.A.; Dixon, D.A. Ionization Potential, Electron Affinity, Electronegativity, Hardness, and Electron Excitation Energy: Molecular Properties from Density Functional Theory Orbital Energies. *J. Phys. Chem. A* **2003**, *107*, 4184–4195.
9. Li, X.; Cai, Z.; Sevilla, M.D. Energetics of the radical ions of the AT and AU base pairs: A density functional theory (DFT) study. *J. Phys. Chem. A* **2002**, *106*, 9345–9351.
10. Foresman, J.B.; Frisch, A. *Exploring Chemistry with Electronic Structure Methods*, 2nd ed.; Gaussian, Inc.: Pittsburgh, PA, USA, 1996; pp. 141–160.
11. Frisch, M.J.; Trucks, G.W.; Schlegel, H.B.; Scuseria, G.E.; Robb, M.A.; Cheeseman, J.R.; Scalmani, G.; Barone, V.; Mennucci, B.; Petersson, G.A. *et al. Gaussian 09, Revision A.02*; Gaussian, Inc.: Wallingford, CT, USA, 2009.
